# Effect of Cross-Section on Low-Temperature Fracture Toughness of Marine Engineering Steel Thick Plate

**DOI:** 10.3390/ma18051015

**Published:** 2025-02-25

**Authors:** Kuan Zheng, Liqin Zhang, Chengyang Hu, Lei Hu, Kaiming Wu

**Affiliations:** 1 Collaborative Innovation Center for Advanced Steels, Wuhan University of Science and Technology, Wuhan 430081, China; zk13277063757@163.com (K.Z.); huchengyang@wust.edu.cn (C.H.); 18356064930@163.com (L.H.); wukaiming@wust.edu.cn (K.W.); 2 Department of Applied Physics, Wuhan University of Science and Technology, Wuhan 430081, China

**Keywords:** cross-section effect, microstructural characterization, low-temperature fracture toughness, crack deflection

## Abstract

The cross-section effect leads to variations in the microstructure and thickness of marine engineering steel thick plates, which may result in delaminated tearing and a reduction in material plasticity and fracture toughness. The microstructural characterization of the matrix and the regions near the impact fracture at different depths by the thickness of the thick plate, as well as the crack propagation mechanism, was investigated. The low-temperature fracture toughness at the surface, quarter-thickness, and center at −40 °C were found to be 204 J, 215 J, and 98 J, respectively. Near the impact fracture, the grains of the test steel exhibit significant deformation. The grains at the surface experience fragmentation, leading to grain refinement and the formation of serrated cracks. At the quarter-thickness, cracks display large-angle deflection, and the deflection at the center is notably reduced, accompanied by an increase in the number of voids. In this study, the influence of the cross-section effect on the fracture toughness of marine engineering steel thick plates is thoroughly investigated. The experimental results provide theoretical guidance for the design and production of thick plates with optimal strength and toughness. This study primarily examines the influence of the cross-section effect on the microstructure and low-temperature fracture toughness of the investigated steel. Furthermore, it not only examines the microstructure of the base metal but also employs electron backscatter diffraction (EBSD) technology to characterize and analyze the microstructure near the crack, thereby exploring the influence of the cross-section effect on the fracture mechanism of the investigated steel.

## 1. Introduction

The ocean covers approximately 71% of the Earth’s surface and contains abundant energy resources, including oil and natural gas. As terrestrial resources become progressively scarcer, the development of oceanic energy has become increasingly critical. Marine transportation vessels and offshore drilling platforms are often required to operate in extremely low temperatures, creating a growing demand for thick, high-strength steel plates with high thickness, low-temperature fracture toughness, and superior welding performance for marine engineering applications [1]. Current thick plate production technologies include static ingots, continuous casting billets, electroslag remelting [2], welded composite slabs, vertical continuous casting, and gradient temperature rolling [3], etc. The choice of production technology is influenced by factors such as production cost, product qualification rate, alloy composition, and the specific steel grades being produced. Reducing production costs and improving production efficiency are key drivers for advancing the steel industry.

During the continuous casting of ultra-thick plates (defined as those with a thickness greater than 60 mm [4]), differences in solubility between alloying elements in the liquid and solid phases inevitably lead to the segregation of alloy elements at the center of the thick plate, causing microstructural inhomogeneity along the thickness direction. Accumulated alloying elements tend to promote the formation of martensite in the center, thereby degrading center fracture toughness [5]. After rolling, the billet undergoes a tempering heat treatment process, which includes quenching followed by high-temperature tempering. However, achieving a consistent quenching and cooling rate throughout the entire thickness direction is challenging.

For a 120 mm marine engineering steel thick plate, a quenching and tempering heat treatment process is applied after rolling [6]. Achieving uniform cooling rates across the entire thickness is inherently challenging. The thick plates experience a gradual reduction in cooling rate from the surface to the center during the quenching process, resulting in microstructural variations along the thickness direction. The surface cools the fastest, predominantly forming martensitic structures; the quarter thickness cools more slowly, resulting in a mixed structure of lath bainite/lath martensite (LB/LM); and the center cools the slowest, forming predominantly bainite (B), which results in a mixture of LB/GB. Consequently, low-temperature fracture toughness varies with these microstructural changes [6]. Thick plates are prone to local delamination, which degrades mechanical properties along the thickness direction, making them susceptible to lamellar tearing during welding or under tensile stresses along the thickness.

Current views on martensite hold that an austenite grain is divided into packets (a group of laths with the same habit plane) and each packet is further subdivided into blocks (a group of laths of the same orientation (same variant)). Morito et al. systematically studied the crystallographic characteristics within LB/LM microstructures, finding that the LB/LM structure comprises a multi-level substructure, including the prior austenite, packet, block, and lath. They observed that small-angle grain boundaries (LAGBs) exist between laths, whereas high-angle grain boundaries (HAGBs) are located between packets and blocks [7]. Zhao and Zhu et al. investigated the low-temperature fracture toughness of bainitic steels and found that prior austenite grain boundaries and packet boundaries within HAGBs can enhance the effective impact absorption energy [8,9]. Cheng and Bhattacharya et al. investigated the low-temperature fracture toughness of low-alloyed high-strength steels and found that coarse carbide particles and micro-segregation significantly degrade the impact fracture toughness of the tested steels [10,11]. Chen et al. investigated the effect of the quenching cooling rate on the microstructural evolution and low-temperature fracture toughness of thick plates across different thicknesses. They found that the density of HAGBs significantly influences the impact fracture toughness at varying thicknesses [12]. Hwang et al. investigated crack propagation behavior in low-carbon bainitic steels at low temperatures, finding that the number of crack deflections was significantly higher in the LM/LB structure than in the GB structure. The HAGBs in LM/LB structures effectively impede crack propagation and enhance impact energy absorption [13]. Wang et al. studied the fracture toughness of 15CrNi3MoV alloy steel, finding that as the quenching cooling rate decreased, the carbide size in the experimental steel gradually increased. The fracture morphology of the steel transitioned from a ductile fracture to a mixed brittle-ductile fracture, and ultimately to a brittle fracture [14].

Numerous previous studies have demonstrated that an increase in the ratio of HAGBs often enhances the low-temperature fracture toughness of the test steel [13,14]. However, in this study, the cross-section effect results in a gradual increase in the proportion of HAGBs from the surface to the center of the thick plate, while the center exhibits the lowest fracture toughness. Therefore, this study systematically elucidates the influence mechanism of the cross-section effect on the low-temperature fracture toughness of the thick plate by analyzing the HAGBs and the microstructure at various positions across the thick plate section.

## 2. Materials and Methods

**Figure 1:**
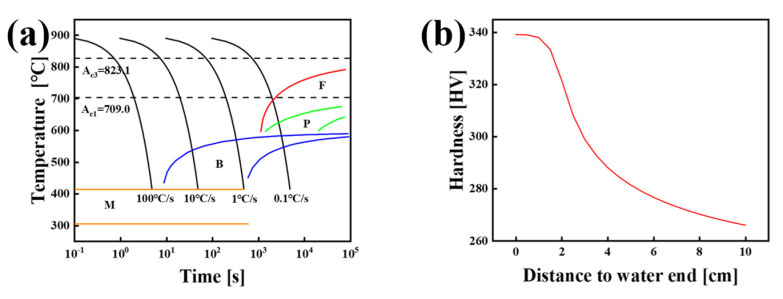
The CCT curve and Jominy curve of the test steel. (**a**) The CCT curve (The red curve represents F (Ferrite); the green curve represents P (Pearlite); the blue curve represents B (Bainite); the orange curve represents M (Martensite); (**b**) The Jominy curve.

The experimental steel is FH690 marine engineering steel, with a thickness of 120 mm, produced industrially by a steel plant, and its chemical composition is provided in Table 1. The thermomechanical processing of the experimental steel involved the following steps: high-quality iron was selected for die casting, and a 380 mm thick billet was heated to 1200 °C and held for 200 min. This was followed by rough rolling at 1200 °C for 8 passes. The opening rolling temperature was 950 °C, and the final rolling temperature was 800 °C. The tempering heat treatment process included quenching at 900 °C followed by tempering at 650 °C. The thermodynamic software JMatPro V13 was employed to calculate the phase transition temperatures of the test steel. The continuous cooling transformation (CCT) curve and the Jominy curve of the test steel were generated, as illustrated in Figure 1. The A_c3_ and A_c1_ temperatures of the experimental steel are 823.1 °C and 709.0 °C, respectively. This study investigates the industrially produced FH690 marine engineering steel thick plate, employing SEM, EBSD, and TEM techniques to characterize the microstructure and fracture mechanism. The carbide size, the proportion of HAGBs, and stress distribution were analyzed statistically.

Microstructural characterization and mechanical property testing were conducted at different thickness locations (surface, quarter, and center) of the test steel. Metallographic samples of the experimental steel were ground, polished, and subsequently etched with a 4% nitric acid ethanol solution. The microstructure was observed using a scanning electron microscope (SEM, Sirion 200) (Thermo Fisher, Waltham, MA, USA). EBSD was used for microstructural characterization, with samples subjected to electro-polishing in a 5% HClO_4_ ethanol solution. The working principle of EBSD analysis involves acquiring a series of Kikuchi patterns through high-energy electron diffraction from the sample surface. Based on the characteristics of the Kikuchi patterns, the interplanar spacing (d) and the angle (θ) between the crystal planes are determined, enabling the identification of the crystal structure, lattice parameters, and orientation relationships between the grains. Kernel average misorientation (KAM) is used to quantify the degree of orientation variation or lattice distortion within grains in the microstructure. Kikuchi band contrast (BC) reflects variations in crystal orientation, enabling the determination of crystal structure and orientation. The EBSD analysis was conducted under the following conditions: acceleration voltage of 20 kV, working distance of 10 mm, tilt angle of 70°, region magnification of 5000×, and a step size of 0.1 µm. Samples were taken from the surface, quarter-thickness, and center of the thick plate, labeled as 0T, 1/4T, and 1/2T, respectively. EBSD characterization of the experimental steel at different locations was performed to obtain crystallographic data. The TEM sample preparation process involved cutting a 3 mm thick sample using a wire-cutting machine, followed by grinding the sample to a thickness of 40–80 μm. Subsequently, a specialized punching machine was used to obtain a 3 mm diameter wafer. Finally, electrolytic double-spray thinning was performed using an MTP-1A electrolytic double-spray instrument. The electrolyte consisted of 10% perchloric acid and 90% acetic acid. The microstructure of the material was analyzed using transmission electron microscopy (TEM, JEM-2010HT) (NULL, Tokyo, Japan).

**Figure 2:**
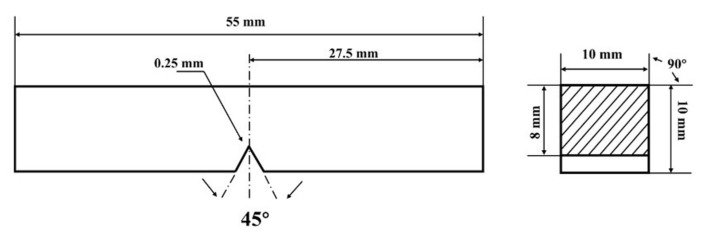
Schematic diagram of the impact sample.

**Figure 3:**
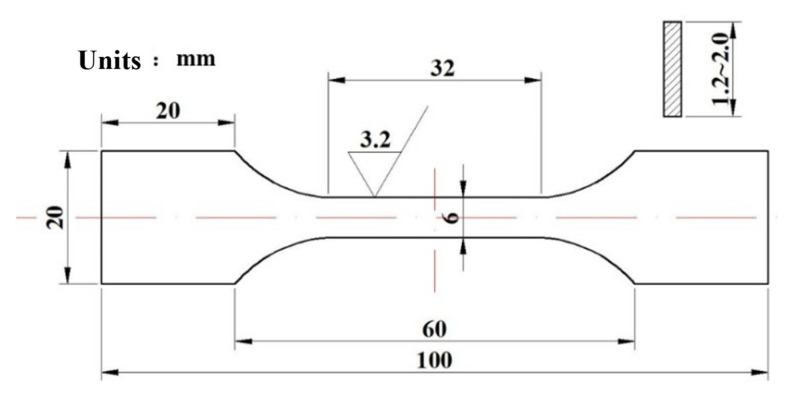
Schematic diagram of the tensile sample.

**Figure 4:**
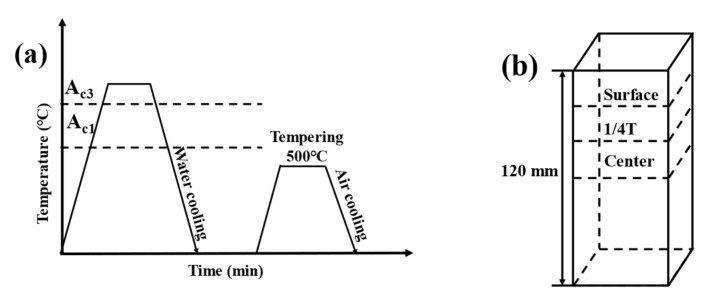
Test steel heat treatment process flow chart and sampling diagram. (**a**) The heat treatment process flow chart (**b**) The sampling diagram.

The inverse standard impact samples measuring 10 mm× 10 mm× 55 mm were prepared and tested using a 450 J pendulum impact tester (WanCe, Shenzhen, China). The impact samples are extracted horizontally at varying thicknesses (0T, 1/4T, and 1/2T) of the thick plate. The direction of the V-shaped notch is aligned parallel to the thickness direction. The impact absorbed energy required for the Charpy impact test is measured by applying a load in the longitudinal direction. The impact tests were performed at a low temperature of −40 °C, with the results presented as the average of three repeated experiments. Tensile samples of the test steel were tested at room temperature using a WDW-100E10kN microcomputer-controlled electronic universal testing machine (WanCe, Shenzhen, China). The static tensile tests were carried out at a strain rate of 10^−3^ s^−1^. The yield strength, tensile strength, elongation, and section shrinkage of the test steel were measured. The impact and tensile samples of the test steel are illustrated in Figure 2 and Figure 3. The heat treatment process flow diagram and sampling schematic in the actual industrial production process are provided in Figure 4.

## 3. Results

### 3.1. Mechanical Properties

The mechanical properties of the experimental steel are provided in Table 2. The 0T sample demonstrates the highest yield strength, tensile strength, and elongation. The yield strength, tensile strength, and elongation of the steel gradually decrease from the surface to the center, with reductions of 31 MPa for yield strength, 45 MPa for tensile strength, and 1% for elongation. Both the 0T and 1/4T samples exhibit excellent low-temperature fracture toughness, with the 1/4T sample demonstrating slightly superior fracture toughness at −40° compared to the 0T sample. The low-temperature fracture toughness of the 1/2T sample is markedly reduced, with the impact energy decreasing by 117 J relative to the 1/4T sample.

### 3.2. Microstructure

**Figure 5:**
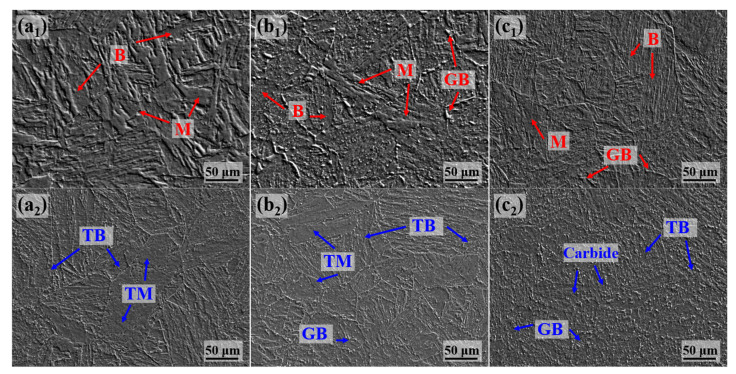
The SEM micrograph of the tested steel. After quenching: (**a_1_**) 0T, (**b_1_**) 1/4T, and (**c_1_**) 1/2T. After tempering: (**a_2_**) 0T, (**b_2_**) 1/4T, and (**c_2_**) 1/2T.

Microstructures of the experimental steel after quenching and tempering at different locations are provided in Figure 5. After quenching, the 0T sample primarily consists of coarse LM, with a small amount of LB present. The microstructure at the quarter-thickness is a mixture of LM and LB, with a small amount of GB present. The microstructure at the center predominantly consists of GB. After high-temperature tempering, high-density dislocations in the LM/LB structure decompose, resulting in tempered martensite (TM) and tempered bainite (TB). Carbon atoms precipitate from the supersaturated solid solution, promoting the nucleation and growth of carbides. Some GB decomposes, resulting in the formation of larger carbides. After tempering, the 0T and 1/4T samples contain only a small amount of carbide precipitation, uniformly dispersed within the TM/TB structure. In contrast, the 1/2T sample exhibits a mixed GB/TB structure after tempering, predominantly composed of GB structures, with large-sized carbides distributed within the bainite matrix.

**Figure 6:**
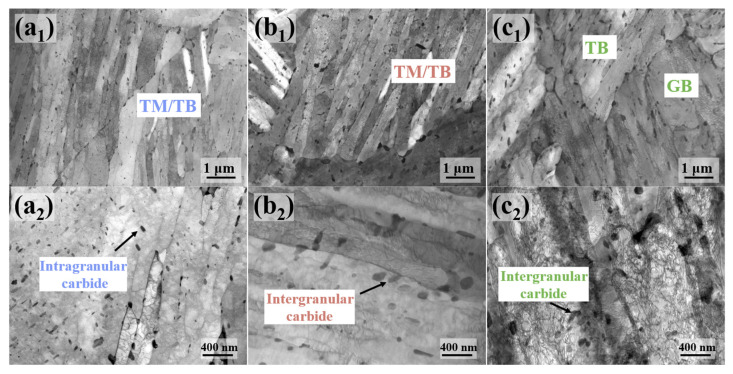
The TEM micrograph of the tested steel. (**a_1_**,**a_2_**) 0T; (**b_1_**,**b_2_**) 1/4T; and (**c_1_**,**c_2_**) 1/2T.

The TEM microstructures of the experimental steel after tempering are presented in Figure 6. The 0T and 1/4T samples exhibit a microstructure consisting of parallel lath structures, whereas the 1/2T sample contains regions with broader lath structures and ferrite. Intergranular and intragranular carbides are present throughout the steel at all locations.

**Figure 7:**
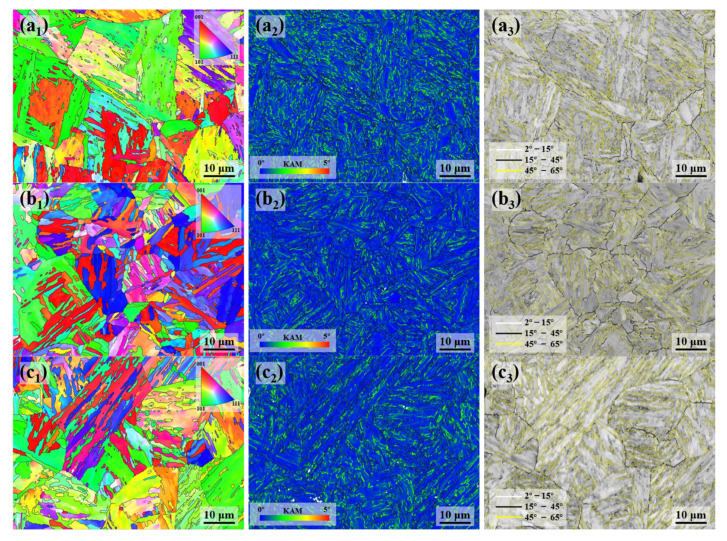
The IPF, KAM, and grain boundary distribution of the test steel. (**a_1_**–**a_3_**) 0T; (**b_1_**–**b_3_**) 1/4T; and (**c_1_**–**c_3_**) 1/2T.

The inverse pole figure (IPF) maps of the experimental steel are presented in Figure 7a_1_–c_1_, with different colors representing various grain orientations [15]. The blocks of the 0T sample are coarser, whereas those in the 1/4T sample are significantly finer. The microstructure at the center of the steel displays some lath structures, along with a ferrite matrix. Within the central region, martensitic phases, resulting from elemental segregation, form fine block structures.

The kernel average misorientation, which reflects the distribution of micro-deformation, was used to evaluate local strain gradients. A higher KAM value indicates a higher concentration of local stress. The stress concentration zones were identified by orientation differences in the range of 0° to 5°, while orientation differences greater than 5° are likely caused by LAGBs and were not considered [16].

The KAM maps of the samples are presented in Figure 7a_2_–c_2_, with the average KAM values of the experimental steel being 0.54°, 0.54°, and 0.5°, respectively. The color gradient in the figure, from blue to red, represents the distribution of local stress, with higher KAM values corresponding to more concentrated stress. The stress distribution is low across all locations, suggesting that the stress in the experimental steel was effectively reduced after tempering.

**Figure 8:**
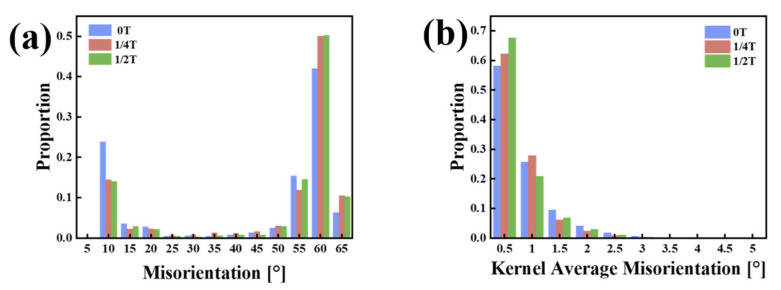
(**a**) Grain boundary statistical distribution. (**b**) KAM statistical distribution of the test steel.

The grain boundary distribution maps of the experimental steel are presented in Figure 7a_3_–c_3_, with white lines representing LAGBs (2–15°) and HAGBs (15–65°) further subdivided into black lines (15–45° range) and yellow lines (45–65° range). The HAGBs in the 15–45° range often correspond to the prior austenite grain boundaries [17]. In the 0T sample, the LM structures are coarser, with some dislocation structures still present, and the dislocations in the martensite may recover, forming a cellular substructure and LAGBs. This results in a higher proportion of LAGBs in the 0T sample than in the 1/4T and 1/2T samples. In contrast, the 1/4T and 1/2T samples have a higher proportion of HAGBs than the 0T sample. The crystallographic information of the test steel was extracted using the AZtecCrystal software. The distributions of grain boundaries and misorientation angles were plotted, and the data were processed to generate the grain boundary distribution and the KAM distribution for the test steel. As shown in Figure 8a, the HAGB ratio of the 1/4T and 1/2T samples is relatively high. As illustrated in Figure 8b, the KAM values of the 0T, 1/4T, and 1/2T samples are low after tempering, indicating that the internal stress of the test steel structure is effectively reduced. The grain boundary of various angle ranges and KAM values of the test steel are summarized in Table 3.

**Figure 9:**
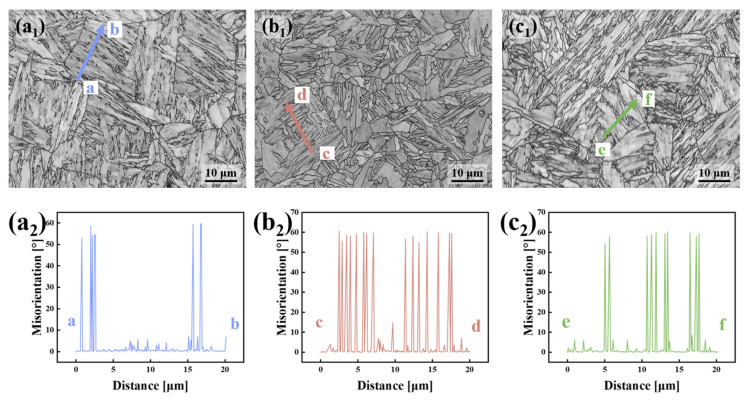
The BC diagram and point-to-point orientation difference of the test steel (The letter a–f represent randomly selected equal-length regions). (**a_1_**,**a_2_**) 0T; (**b_1_**,**b_2_**) 1/4T; and (**c_1_**,**c_2_**) 1/2T.

Figure 9a_1_–c_1_ display the band contrast maps of the test steels at various locations [18]. In these BC maps, equal-length line segments were randomly chosen, and the point-to-point misorientation distribution along these segments was measured, as shown in Figure 9a_2_–c_2_. To evaluate the ability of the test steel’s microstructure at various thicknesses to hinder crack propagation, a 20 μm long line segment was selected in the BC maps to observe variations in orientation differences within this range. The misorientation within a 20 μm range was measured within the grains of the microstructure. The point-to-point misorientation distribution maps clearly indicate that the microstructure between HAGBs is predominantly composed of LAGBs. The 1/4T sample exhibits a significantly higher peak in the misorientation distribution compared to the 0T and 1/2T samples, indicating a greater density of HAGBs between points c and d. Consequently, when a crack propagates through the 1/4T sample, it is more likely to experience multiple deflections, thereby dissipating more energy. In the 0T sample, the blocks are coarser, and within these blocks, LAGBs dominate, leading to a marked reduction in peak values between points a and b. In the 1/2T sample, several M/A islands are observed between points e and f, resulting in multiple peaks in the distribution. The large M/A islands can significantly impair the low-temperature fracture toughness of the steel by inducing crack initiation and propagation along these boundaries.

**Figure 10:**
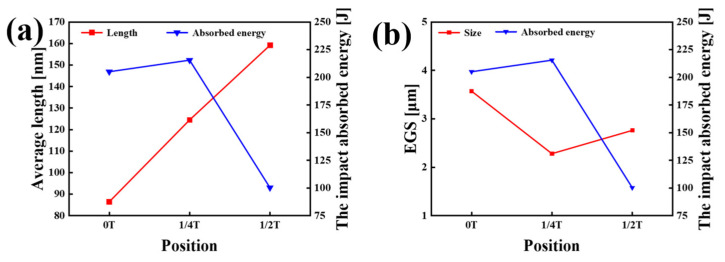
Test steel. (**a**) Relationship between carbide size and the low-temperature fracture toughness of the test steel. (**b**) Relationship between the EGS and low-temperature fracture toughness of tested steel.

The HAGBs partition the microstructure into regions, which are defined as the effective grain size (EGS) [19]. The EGS is derived from EBSD data processed using the crystallography software AZtecCrystal 2.1.2. The EGS values at various thickness positions were found to be 3.57 μm, 2.28 μm, and 2.76 μm, respectively. The relationship between the average carbide size, EGS, and fracture toughness is shown in Figure 10.

### 3.3. Impact Properties

**Figure 11:**
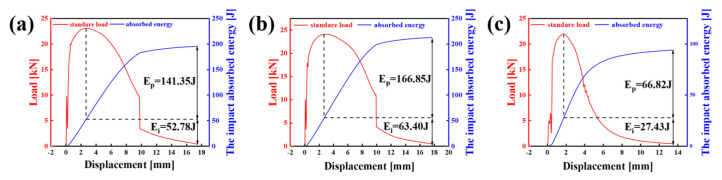
Relationships between impact load and displacement during impact tests of the test steel at −40 °C (the blue curve is the relationship between impact energy and displacement, and the red curve is the relationship between loading force and displacement The dashed lines distinguish the E_i_ and E_p_). (**a**) 0T, (**b**) 1/4T, and (**c**)1/2T.

Charpy impact tests were conducted on the experimental steel at 0T, 1/4T, and 1/2T at −40 °C, and the resulting load–displacement curves are presented in Figure 11. During the process of impact fracture, the energy (E) absorbed by the sample during fracture consists of the crack initiation energy (E_i_) and the crack propagation energy (E_p_). E_i_ represents the energy absorbed by the sample as it deforms and undergoes work, hardening until reaching the maximum impact force (F_max_). E_p_ represents the energy absorbed from the initiation of the crack until the failure [20,21,22]. From the impact curves, it is evident that the E_i_ and E_p_ values for the 0T, 1/4T, and 1/2T samples are 52.78 J, 63.40 J, and 27.43 J and 141.35 J, 166.85 J, and 66.82 J, respectively. The E_i_ and E_p_ values for the 0T and 1/4T samples are significantly higher than those for the 1/2T sample, suggesting that the central region of the thick plate is more prone to crack initiation and fracture under loading.

### 3.4. Impact Fracture Morphology and Microstructure in the Vicinity of Cracks

**Figure 12:**
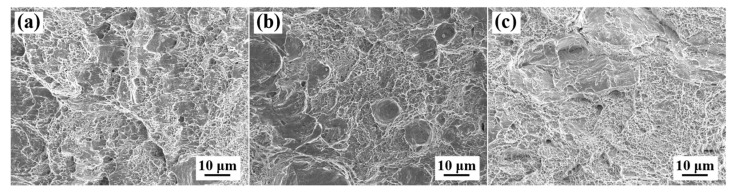
Impact fracture morphology of the test steel. (**a**) 0T; (**b**) 1/4T; and (**c**) 1/2T.

Following the Charpy impact test, the fracture surface and the microstructure near the crack were characterized using microscopy. Figure 12 presents the SEM morphology of the impact fracture surfaces of the 0T, 1/4T, and 1/2T samples. The 0T samples exhibit typical ductile fracture characteristics, with fracture morphologies featuring dimples and tear ridges. The surface primarily consists of martensite, and the fracture morphology displays fine dimples. The 1/4T sample exhibits typical ductile fracture characteristics. The quarter-thickness region contains a mixed structure of LM/LB, where larger and deeper dimples are formed, indicating superior fracture toughness. In contrast, the 1/2T sample exhibits smaller dimples and larger cleavage surfaces. The center region exhibits more large cleavage planes, with a few small dimples, displaying a quasi-cleavage fracture morphology.

To further investigate the mechanism of crack propagation and its relationship with the surrounding microstructure, EBSD characterization of crack propagation near the impact fracture was conducted. Samples that underwent the Charpy impact test were collected and sampled along the direction perpendicular to the V-shaped notch at the fracture. The crack direction was observed using an optical microscope, and the crack path was marked. Following electrolytic polishing, EBSD analysis was performed to obtain crystallographic information on the crack and the microstructure near the fracture.

**Figure 13:**
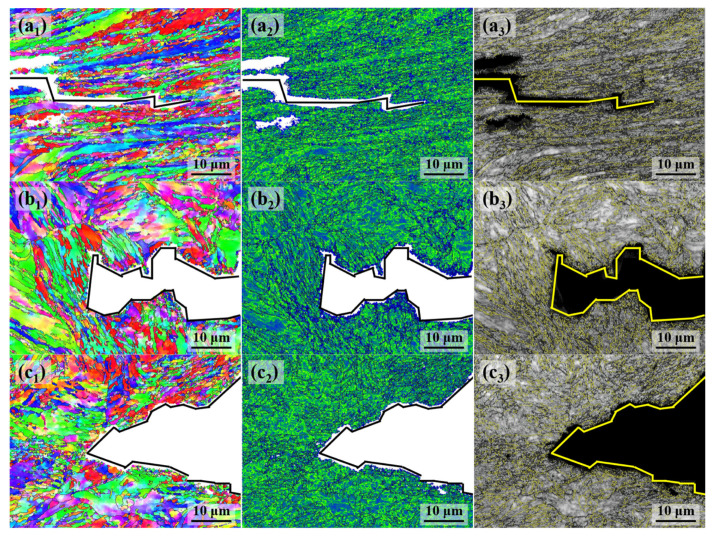
The IPF, KAM, and grain boundary distribution near the fracture surface of the test steel. (**a_1_**–**a_3_**) 0T; (**b_1_**–**b_3_**) 1/4T; and (**c_1_**–**c_3_**) 1/2T.

Figure 13 illustrates the morphology and deflection of the crack. Observations of the primary cracks in samples from different positions of the thick plate reveal that cracks in the surface exhibit a serrated morphology. In the quarter-thickness and center, cracks exhibit significant deflections, primarily occurring at HAGBs. Surface cracks experience fewer deflections, while quarter-thickness cracks show noticeably more deflections, primarily involving large-angle deflections. Cracks in the center also experience fewer deflections, with deflection angles that are significantly smaller than those observed in the quarter-thickness region. Figure 13a_1_–c_1_ show the IPF maps of crack propagation at various locations. After low-temperature impact testing, the surface exhibits noticeable deformation, with grains aligned in the same direction and significant grain fragments, leading to grain refinement. In the quarter-thickness and center regions, only small grains are present near the cracks, showing partial grain fragments. Figure 13a_2_–c_2_ present the KAM maps of the microstructure surrounding the cracks. Based on the statistics, the KAM values surrounding the cracks for the 0T, 1/4T, and 1/2T samples are 0.89°, 0.95°, and 1.02°, respectively. After the impact test, the thick plate steel samples from different regions underwent significant plastic deformation, resulting in an increase in the KAM values.

**Figure 14:**
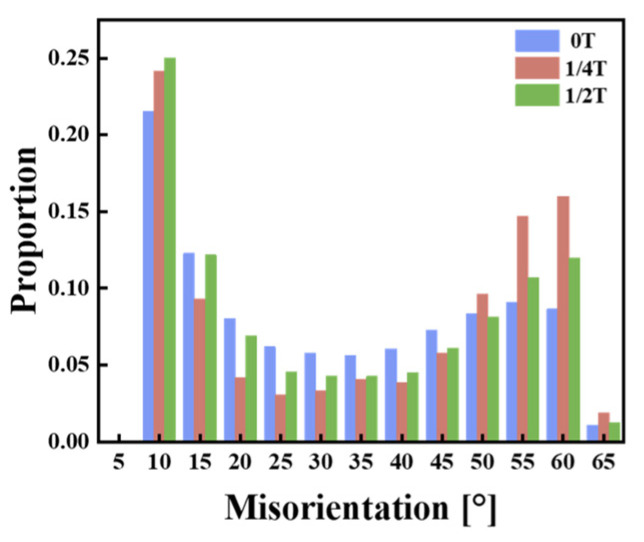
The size angle grain boundary distribution map at the fracture of the test steel.

The distribution maps of HAGBs for the thick plate samples at various positions are shown in Figure 13a_3_–c_3_. Statistical analysis of the HAGBs surrounding the cracks in the samples is provided in Figure 14, with the results summarized in Table 4. Compared with the distribution of HAGBs in the test steel before the impact test, it is observed that the percentage of HAGBs in the 45~65° range decreases, while the percentage in the 15~45° range increases. Specifically, the 0T sample shows the largest change in the 15~45° range, increasing from 5.68% to 39.2%.

## 4. Discussion

### 4.1. Effect of Microstructure and Carbides on Low-Temperature Fracture Toughness

The quenching cooling rate for quenched and tempered thick plate steel used in marine engineering typically ranges from 1 °C/s to 30 °C/s, with the cooling rate decreasing from the surface to the center. From the CCT curve shown in Figure 1a, it can be inferred that an M/B mixed structure will form during the quenching and cooling process of the marine engineering steel thick plate. Analysis of the Jominy curve (Figure 1b) indicates that the hardness of the test steel reaches 260 HV after quenching. Combined with SEM images of the microstructure, it is concluded that the thick plate has fully transformed into a martensitic and bainitic structure. During quenching, the surface cools at the fastest rate, primarily resulting in the formation of the LM structure. As the cooling rate decreases from the surface to the one-quarter thickness, a mixed LM/LB structure forms. With increasing thickness, the cooling rate slows further at the center, where the austenite phase remains for a longer period. As a result, LB gradually decreases and GB gradually increases, with the center predominantly containing the GB structure [23]. Studies have shown that the substructure of both LM and LB consists of lath structures, whereas GB consists of a ferrite matrix with M/A islands [24]. Simultaneously, the continuous casting process gives rise to a degree of elemental enrichment within the center of the thick plate. The localized aggregation of carbon content induces an elevation in the tempering stability of some austenite and an augmentation in hardenability, thereby resulting in the formation of a partially martensitic hard-phase microstructure in the center [25].

After high-temperature tempering, the supersaturated carbon in the martensite precipitates, forming fine carbides. The lattice distortion in the martensite is gradually eliminated, the dislocation density decreases, and the martensite transforms into ferrite. In the upper bainite, the carbides in the bainite ferrite coarsen further, the ferrite undergoes recovery and recrystallization, the dislocation density decreases, and the grain size increases. The carbides in the lower bainite also grow; however, due to their dispersed nature, fine carbides are predominantly formed after high-temperature tempering [26,27,28,29]. During tempering, the LM and LB structures decompose, and the precipitated carbides are relatively fine in size. The M/A islands in the GB structure, which have a higher carbon content, are typically of micron size. Larger M/A islands promote the formation and growth of carbides, resulting in larger carbide sizes upon decomposition. After tempering, a number of relatively large-sized carbides are present in the tempered structure of the quarter section and the central part.

**Figure 15:**
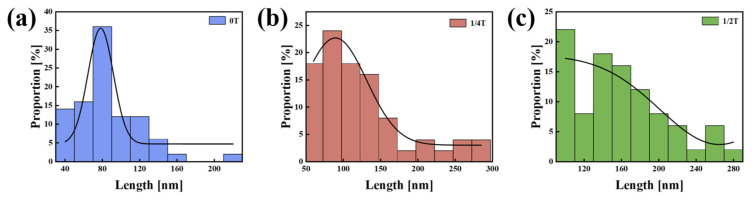
Histogram of the carbide sizes of the test steel at different positions. (**a**) 0T; (**b**) 1/4T; and (**c**) 1/2T. (The curve represents the grain size distribution trend).

All carbide sizes were statistically analyzed in 100 TEM images, and the results are presented in Figure 15. The average carbide sizes in the 0T, 1/4T, and 1/2T test steels were 86.44 ± 2.15 nm, 124.46 ± 2.59 nm, and 159.12 ± 3.44 nm, respectively. The carbide size gradually increases from the surface to the center of the thick plate. Larger carbides typically serve as crack initiation sites, which can lead to the propagation of cleavage cracks. Moreover, the larger the carbide size, the more easily cracks initiate, which reduces the fracture toughness of the material [30]. The presence of larger carbides significantly diminishes the fracture toughness of the center region.

The initiation of cleavage cracks is associated with the presence of carbides and non-metallic inclusions. Under stress, carbides and non-metallic inclusions can generate microcracks that induce crack initiation and propagation. The stress required for cleavage fractures can be calculated by modifying the Griffith criterion. This model provides the relationship between local decohesion fracture stress and carbide sizes [31].

(1) $$\sigma_{yy}={\left[\frac{\pi E\gamma_{p}}{\left(1-\nu^{2}\right)d}\right]}^{1/2}$$

In this equation, σ_yy_ represents the critical local cleavage fracture stress, E denotes Young’s modulus (generally considered to be 200 GPa for high-strength low-alloy steel), γ_p_ indicates the effective surface energy (generally considered to be 7 J/m^2^ for the interface between ferrite and carbide), ν is Poisson’s ratio (generally considered to be 0.29), and d represents the carbide size. According to Griffith’s equation, the critical local cleavage fracture stress is inversely proportional to the carbide size. Nanomeasure software was used and the average value was calculated. The average carbide size was then used to determine the local dissociation fracture stress of the test steel at various positions of thickness. By substituting average values of carbide sizes of different thicknesses (0T, 1/4T, and 1/2T), the local decohesion fracture stresses for the surface, quarter-thickness, and center positions of the samples are calculated to be 7.45 GPa, 6.21 GPa, and 5.49 GPa, respectively. As the carbide size increases, the critical local decohesion fracture stress decreases, rendering the center of the test steel more susceptible to cleavage fracture under impact loading. Bing et al. observed that when a crack propagates into the matrix of the samples and encounters small carbide particles, it undergoes a process of blunting, leading to stress relief. As the crack propagates further, plastic deformation of the matrix occurs, and microvoids form at the sites of small carbide particles. The accumulation of these microvoids results in the formation of dimples, leading to ductile fracture [32].

After tempering, most of the GB in the sample decomposes, although a small amount of M/A components remain. As shown in Figure 5a_2_–c_2_, a portion of the GB structure persists in the 1/4T and 1/2T samples after tempering. A statistical analysis of 100 SEM images of the tempered 1/4T and 1/2T samples reveals that the proportion of M/A components in the quarter-thickness and center regions is 1.43% and 7.84%, respectively. During the formation of GB, carbon enrichment results in M/A components having a higher hardness, creating a significant hardness mismatch with the bainitic ferrite matrix. It is generally believed that M/A islands in GB induce quasi-cleavage fractures, significantly reducing the fracture toughness of the steel [33].

At the quarter-thickness position, M/A is mainly distributed in point-like and short-rod forms, whereas in the center region, M/A components predominantly take on long-rod and block-like shapes. Under stress, the bainitic ferrite matrix undergoes plastic deformation, generating internal stress near the M/A components, and the interface between the two experiences substantial stress concentration. This results in the cracking of block-like M/A components or debonding of the M/A components from the ferrite matrix, generating microcracks and leading to cleavage fracture [34].

After tempering, the center region still contains some blocky/rod-like M/A components. The large-size carbides and the large volume of M/A induce the initiation of microcracks, thereby reducing the fracture toughness of the center region.

### 4.2. Effect of a High-Angle Grain Boundary on the Fracture Toughness of Test Steel

It is generally believed that HAGBs impede crack propagation and enhance the low-temperature fracture toughness of the specimen. During fracture, when cracks encounter HAGBs, they experience significant deflections. The greater the number of HAGBs, the more times the crack changes direction, consuming more energy during crack propagation [35]. The statistical diagrams of HAGBs and KAM for the samples are shown in Figure 8, with numerical values summarized in Table 3. The proportion of LAGBs in the 0T sample is higher than in the 1/4T and 1/2T samples, while the proportion of HAGBs in the 0T sample is lower than in the 1/4T and 1/2T samples. In the 0T sample, the LM blocks are coarser, and LAGBs dominate within these coarse blocks. This results in a significantly higher proportion of LAGBs in the 0T sample compared to the 1/4T and 1/2T samples. At the quarter-thickness position, during quenching, LB forms preferentially, segmenting the prior austenite grains. The martensitic blocks formed during the phase transformation then grow within these segmented regions. The growth of martensitic blocks is constrained, leading to smaller block units and a finer microstructure [36]. The block units between these martensitic laths primarily form HAGBs, which increases the proportion of HAGBs at the 1/4T position compared to the 0T sample. At the center position, the microstructure consists primarily of GB, where M/A islands form HAGBs with the bainitic ferrite matrix, while LAGBs are formed within the ferrite matrix [19,37,38,39]. The presence of alloy element segregation in the center results in the formation of some hard-phase martensite, which generates HAGBs [26]. Additionally, M/A islands persist in the center region, contributing to the higher proportion of HAGBs in the 1/2T sample. Therefore, the higher proportion of HAGBs at the 1/4T position increases the fracture toughness, while the higher HAGB proportion at the center is associated with the hard-phase martensite and remaining M/A islands, which results in lower fracture toughness.

From the surface to the center of the thick plate, the cooling rate varies, resulting in a gradual reduction in martensitic content and an increase in bainitic content. Coarse martensite blocks form at the surface, while more GB structures develop in the center, causing an initial decrease in EGS followed by an increase towards the center [39]. A comparison of Figure 10a,b indicates that there is no strict proportional or inverse relationship between the average carbide size, EGS, and the impact fracture toughness of the specimen steel. It can be inferred that low-temperature fracture toughness is the combined result of both factors. Large carbide particles tend to induce stress concentration, resulting in micro-crack initiation, which reduces crack initiation resistance. Conversely, smaller carbide particles and a smaller EGS inhibit crack propagation by dissipating energy during crack growth, thereby improving the crack propagation resistance.

**Figure 16:**
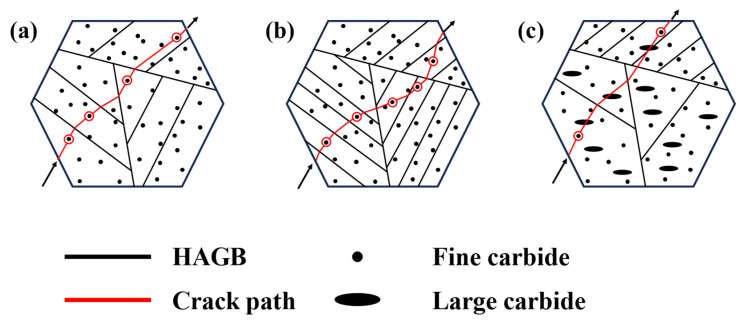
Schematic diagram of crack propagation in the test steel. (**a**) 0/T; (**b**) 1/4T; and (**c**) 1/2T. (The arrow represents the direction of crack propagation. The red circles represent that the stress gets released).

The crack propagation diagram for the tested steel is shown in Figure 16. In this diagram, black lines represent HAGBs, red lines denote the crack propagation paths, dots represent small carbide particles, and ellipses represent larger carbides. In Figure 16a, for the 0T sample, the effective grain size (EGS) is larger, leading to fewer crack deflections and facilitating stress release at smaller carbides. In Figure 16b, for the 1/4T sample, the EGS is smaller, resulting in more crack propagation events and higher energy consumption during propagation. In Figure 16c, for the 1/2T sample, there are more large carbides. When the crack propagates to a large carbide, stress cannot be effectively released, and voids can form between the carbide and the matrix, causing the crack to propagate along these voids. In the various regions of the thick plate, the energy consumed during crack propagation is significantly lower in the center compared to the surface and quarter-thickness regions. As a result, the fracture toughness of the thick plate steel significantly decreases in the center, making larger cracks more likely to form.

### 4.3. Crack Initiation and Propagation

Under impact loading, cracks in the standard impact samples of the test steel initially form at the V-shaped notch. The displacement at the bottom of the notch is largest, and stress concentration is the most severe, leading to crack initiation at this location. Once the crack initiates, its tip becomes a new site of stress concentration, and the remaining impact energy results in concentrated plastic deformation and cracking near the crack tip, ultimately causing the fracture of the test steel. Under impact loading, the applied impact energy is very high, and stress concentration at the crack tip is extremely severe. During this stage, the number of potential dislocation sources near the crack tip increases significantly, with each dislocation source potentially emitting a large number of dislocations, forming severe dislocation pile-ups [40].

In surface specimens, the predominant microstructure is quenched martensite, with a high dislocation density. During the impact process, dislocations generated from dislocation sources and the high-density dislocations in the grain contribute to the formation of high-density dislocation walls and entanglements. These dislocation walls and entanglements gradually transform into LAGBs, which, through continued dislocation absorption, eventually evolve into HAGBs within the 15~45° range [30]. As a result, the proportion of HAGBs in the 15~45° range increases significantly in the 0T sample. The primary grain boundaries hindering crack propagation are those in the 45~65° range, while the 15°~45°HAGBs exert a weaker effect on crack propagation [30].

In the quarter-thickness region, the tempered structure consists of LM/LB, with a higher density of HAGBs. Under stress, cracks deflect at HAGBs, consuming more energy, and only small grains form near the crack tip. In the center region, the microstructure primarily consists of GB, and significant stress concentrations exist between the matrix and MA islands, appearing as more voids in the EBSD maps. The matrix predominantly consists of LAGBs, which exert a weaker effect on inhibiting crack propagation. As a result, cracks in the center region experience smaller deflection angles compared to those in the quarter-thickness region, resulting in the lowest fracture toughness.

By consulting the extensive literature and substituting laboratory measurements into empirical formulas, the stress intensity factor and the J-integral of the test steel were determined [41,42,43]. The calculation results are shown in Table 5. The calculation results indicate that, compared to the 1/2T test steel, the 0T and 1/4T test steels exhibit superior fracture toughness.

## 5. Conclusions

In this study, the influence of the cross-sectional effect on the microstructure and fracture toughness of marine engineering steel thick plates at varying thicknesses at −40 °C is analyzed. This study aims to provide a theoretical basis for optimizing the composition design and production process of steel plates, with a detailed understanding of how the microstructure affects crack initiation and propagation under impact load. Based on the experimental results, the following conclusions are drawn.

1. After quenching and tempering, the marine engineering steel thick plates exhibit pronounced microstructural differences across various positions due to the cross-section effect. The surface microstructure predominantly consists of tempered martensite, whereas the microstructure at the quarter-thickness region is a mixture of tempered martensite and tempered bainite. The center microstructure primarily consists of granular bainite. The fracture toughness of the center is markedly lower than that of the surface and quarter-thickness regions. The impact energies measured at different locations are as follows: 205 J for the surface, 215 J for the quarter-thickness region, and 100 J for the center.
2. Both the density of HAGBs and carbide size after tempering have a significant impact on the fracture toughness of the test steel. Larger carbides tend to cause stress concentration, which can initiate cracks, whereas smaller carbides can release stress during crack propagation, thereby enhancing fracture toughness. In the quarter-thickness region, the higher density of HAGBs plays a crucial role in deflecting cracks during propagation, thereby dissipating energy and enhancing the fracture toughness of the steel.

In subsequent studies, finite element simulations will be employed to investigate the influence of carbides and grain boundaries on crack initiation and propagation under stress. This study focuses solely on the size of carbides and does not examine other precipitates, which introduces certain limitations. The strength and toughness of marine engineering steels are strongly correlated with service temperature. Potential future research directions include the influence of the section effect on the brittle–ductile transition temperature and fracture toughness of marine engineering steels at extremely low temperatures (−196 °C). Research will also explore the effects of seawater corrosion resistance and welding performance on the fracture toughness of thick plates.

## Figures and Tables

**Table 1:** Chemical composition of the test steel [wt%].

C	Mn	Si	S	P	Cr	Ti + V	Fe
0.115	1.51	0.32	0.003	0.006	0.51	0.02	Bal

**Table 2:** Mechanical properties of the test steel.

Position	Tensile Strength [MPa]	Yield Strength [MPa]	Total Elongation [%]	Impact Absorbed Energy [J]	Average Value [J]
0T	785 ± 5	718 ± 5	20.0 ± 0.4	221.5, 194.1, 199.3	204.9 ± 16.6
1/4T	774 ± 8	697 ± 9	19.5 ± 0.1	204.0, 212.0, 230.2	215.4 ± 14.8
1/2T	755 ± 5	673 ± 8	19.0 ± 0.3	106.3, 93.9, 96.4	98.8 ± 7.5

**Table 3:** Statistics of proportion of grain boundaries and KAM.

Position	Proportion of Grain Boundary [%]			KAM [°]
	5~15	15~45	45~65	
0T	33.70	5.68	60.60	0.54
1/4T	20.60	7.24	72.20	0.54
1/2T	19.10	4.89	76.00	0.50

**Table 4:** Statistics of proportion of grain boundaries and KAM of the microstructure near the fracture.

Sample	Proportion of Grain Boundary [%]			KAM [°]
	5~15	15~45	45~65	
0T	32.4	39.2	28.4	0.89
1/4T	32.8	23.7	43.4	0.95
1/2T	35.9	30.5	33.5	1.02

**Table 5:** Calculation results of the K_IC_ and J-integral.

Position	K_IC_ [kJ/m^2^]	J-Integral [MPa·m^1/2^]
0T	221.87	225.43
1/4T	227.49	236.99
1/2T	154.07	108.71

## Data Availability

The original contributions presented in this study are included in the article.
